# Poor balance and lower gray matter volume predict falls in older adults with mild cognitive impairment

**DOI:** 10.1186/1471-2377-13-102

**Published:** 2013-08-05

**Authors:** Hyuma Makizako, Hiroyuki Shimada, Takehiko Doi, Hyuntae Park, Daisuke Yoshida, Kazuki Uemura, Kota Tsutsumimoto, Teresa Liu-Ambrose, Takao Suzuki

**Affiliations:** 1 Section for Health Promotion, Department for Research and Development to Support Independent Life of Elderly, Center for Gerontology and Social Science, National Center for Geriatrics and Gerontology, 35 Gengo, Morioka-machi, Obu, Aichi 4748511, Japan; 2 Japan Society for the Promotion of Science, Tokyo, Japan; 3 Section for Physical Functioning Activation, Department of Functioning Activation, Center for Gerontology and Social Science, National Center for Geriatrics and Gerontology, Obu, Aichi, Japan; 4 Aging, Mobility, and Cognitive Neuroscience Laboratory, Department of Physical Therapy, University of British Columbia, Vancouver, BC, Canada; 5 Brain Research Centre, University of British Columbia, Vancouver, BC, Canada; 6 Centre for Hip Health and Mobility, Vancouver Coastal Health Research Institute, University of British Columbia, Vancouver, BC, Canada; 7 Research Institute, National Center for Geriatrics and Gerontology, Obu, Aichi, Japan

## Abstract

**Background:**

The risk of falling is associated with cognitive dysfunction. Older adults with mild cognitive impairment (MCI) exhibit an accelerated reduction of brain volume, and face an increased risk of falling. The current study examined the relationship between baseline physical performance, baseline gray matter volume and falls during a 12-month follow-up period among community-dwelling older adults with MCI.

**Methods:**

Forty-two older adults with MCI (75.6 years, 43% women) underwent structural magnetic resonance imaging and baseline physical performance assessment, including knee-extension strength, one-legged standing time, and walking speed with normal pace. ‘Fallers’ were defined as people who had one or more falls during the 12-month follow-up period.

**Results:**

Of the 42 participants, 26.2% (n = 11) experienced at least one fall during the 12-month follow-up period. Fallers exhibited slower walking speed and shorter one-legged standing time compared with non-fallers (both *p* < .01). One-legged standing time (sec) (standardized odds ratio [95% confidence interval]: 0.89 [0.81, 0.98], *p* = .02) was associated with a significantly lower rate of falls during the 12-month follow-up after adjusting for age, sex, body mass index, and history of falling in the past year at baseline. Voxel-based morphometry was used to examine differences in baseline gray matter volume between fallers and non-fallers, revealing that fallers exhibited a significantly greater reduction in the bilateral middle frontal gyrus and superior frontal gyrus.

**Conclusions:**

Poor balance predicts falls over 12 months, and baseline lower gray matter densities in the middle frontal gyrus and superior frontal gyrus were associated with falls in older adults with MCI. Maintaining physical function, especially balance, and brain structural changes through many sorts of prevention strategies in the early stage of cognitive decline may contribute to decreasing the risk of falls in older adults with MCI.

## Background

Falls and fall-related injuries are a common healthcare problem, and represent important causes of morbidity and mortality in older populations. One-third of all community-dwelling adults age 65 years and older experience at least one fall annually [1]. Many distinct causes for falls in older people have been reported by a large number of studies [1-4]. Impaired physical function, particularly muscle weakness and problems with gait and balance, are the most important contributors to the risk of falling [5]. The ageing of the worldwide population in recent decades has resulted in an increasing number of older adults with cognitive decline [6], and cognitive impairment has also been found to increase the risk of falling [7-10]. As such, correctly identifying the risk factors for falling among older adults with cognitive impairment is an important research question. In addition, people with cognitive impairment recover less well after a fall than those without cognitive impairment [11]. Therefore, the falling may have negative impact on health in older people with cognitive impairment compare with those without cognitive impairment. In older individuals with mild cognitive impairment (MCI) in particular, consideration of a broad range of causes of falls could play a role in reducing the fall risk and providing strategies to prevent falls among the high-risk population.

Several studies have examined falling in older adults with dementia, such as Alzheimer’s disease [11,12]. However, little research has focused on falling among people with MCI, even though mild declines in cognitive function have been reported to be an important factor associated with falling [13]. Liu-Ambrose et al. demonstrated that older community-dwelling people with MCI but not dementia were at greater risk of falling than those without MCI [14]. Brain structural changes represent one of the key clinical features associated with MCI, including gray matter volume loss [15] and white matter hyperintensities (WMH) [16]. A recent prospective study indicated that greater WMH burden predicts falls over 12 months in non-demented community-dwelling older adults [17].

Although prospective evidence suggests that WMH are an important risk factor for falls in community-based older populations [17,18], it remains unclear whether gray matter volume predicts falls and which regions are related to a greater risk of falls in older adults with MCI. Structural changes in the brain have been linked to motor performance deficits [19]. WMH was reported to exhibit a negative correlation with postural stability involved balance, stepping and gait [20], while reduced gray matter density is associated with impaired gait performance [21-23] and postural instability [24]. Kido et al. [24] suggested that postural instability is associated with gray matter volume loss, and is related to pathological cognitive decline, such as MCI and AD. Lower gray matter volume has been found to be related not only to cognitive decline, but also to decreased physical function. Thus, gray matter volume loss may increase the risk of falls in older adults with MCI. In particular, a smaller volume of the prefrontal area might be associated with poor physical performance [22,23], such as slower gait and poor balance, but no evidence has been reported that smaller brain volume of specific regions is related to the occurrence of subsequent falls in older adults with MCI. In the current study, we sought to examine whether physical performance and gray matter volume were related to falls during a 12-month follow-up period among community-dwelling older adults with MCI.

## Methods

### Participants

The sample for this longitudinal study consisted of 42 community-dwelling older adults with MCI who completed a randomized controlled trial (RCT) (trial registration: UMIN-CTR UMIN000003662) evaluating the effects of multicomponent exercise on cognitive function. The Ethics Committee of the National Center for Geriatrics and Gerontology approved the study protocol. The study design and the primary results of the RCT have been described previously [25]. All participants gave written informed consent prior to taking part in the study. Briefly, participants enrolled in the RCT were: aged 65 years and over, community dwelling, and did not suffer from dementia. All participants met the Petersen criteria for MCI [26]. Participants who had a Clinical Dementia Rating (CDR) = 0, or a CDR of 1–3, a history of neurological, psychiatric, or cardiac disorders or other severe health issues, use of donepezil, impairment in basic activities of daily living (ADL), and participation in other research projects were excluded from the RCT study. A total of 100 participants took part in the RCT and completed neuropsychological assessments including language, memory, attention, and executive function tests. All subjects in this study had objective impairments at least 1.5 standard deviations below the age-adjusted mean for at least one of the neuropsychological tests. The participants were classified to an amnestic MCI (aMCI) group (n = 50) with neuroimaging measures, and other MCI group (n = 50) before the randomization. The subjects in each group were then randomly assigned to either a multicomponent exercise group or an education control group using a ratio of 1:1. The sample for this longitudinal study involved participants in a control group. Of the 50 participants in the control group, 42 completed fall follow-up assessments during the 12-month follow-up period.

### Physical performance measures

At baseline, all participants underwent an extensive assessment of measures by licensed and well-trained physical therapists.

### Knee-extension strength

Isometric knee extension strength was tested twice using a dynamometer (Model MDKKS, Molten Co Ltd, Hiroshima, Japan) from the dominant leg (self-reported side they would use to kick a ball as far as possible). Knee extension was measured while the participant was sitting on a chair with a backrest and the knee flexed to 90°. A testing pad was attached to the front lower leg of the participant and strapped to the leg of the chair. The participant was instructed to push the pad with maximal strength. Licensed and well-trained physical therapists confirmed compensatory movement and assessed muscle strength. Participants practiced several times before data collection. Two trials were conducted, and the maximal isometric strength was determined as the peak torque (Nm) in the data analysis.

### One-legged standing (OLS) test

The OLS test is a commonly used balance assessment of postural stability. For the OLS test, we asked participants to look straight ahead at a dot 50 cm in front of them, then to stand on their preferred leg with their eyes open and hands down alongside the trunk. OLS balance was measured as the length of time (0–60 s) participants were able to stand on one leg. The better of the two trials was used for statistical analysis.

### Walking speed

WS was measured using a 5-m walking test. The participants’ usual WS was measured over an 11-m straight and level path. The time taken (in seconds) to pass the 5-m mark on the path was used as the participant’s score. A 3-m approach was allowed before the starting marker, and an additional 3 m of space was provided after the end marker of the 5-m path to ensure a usual walking pace throughout the task. Participants were instructed to walk the 11-m path at their usual walking pace. The time to complete the 5-m walking test was measured once and was used to calculate walking speed (m/min).

### Falls follow-up

Fall frequency during the 12-month follow-up period was measured with two face-to-face interviews at 6 months and 12 months after baseline measurements. A fall was defined as “an unexpected event in which the person comes to rest on the ground, floor, or lower level” [27]. In this study, ‘fallers’ were defined as people who had at least one fall during the 12-month follow-up period [28].

### Magnetic resonance imaging (MRI) procedure

Magnetic resonance imaging (MRI) was performed using a 1.5-T system (Magnetom Avanto, Siemens, Germany). Three-dimensional volumetric acquisition of a T1-weighted gradient-echo sequence was then used to produce a gapless series of thin sagittal sections using a magnetization preparation rapid-acquisition gradient-echo sequence (repetition time, 1,700 ms; echo time, 4.0 ms; flip angle 15°, acquisition matrix 256 × 256, 1.3-mm slice thickness). Tissue segmentation, regulation, registration, and normalization were conducted in the VBM8 toolbox (http://dbm.neuro.uni-jena.de/vbm/), which is incorporated in the SPM8 software (http://www.fil.ion.ucl.ac.uk/spm/), running on MATLAB R2010a (Mathworks). Diffeomorphic Anatomical Registration using Exponentiated Lie algebra (DARTEL) [29] was conducted for the image analysis. The normalized images were transformed into Montreal Neurological Institute space. The gray matter images were then smoothened using a Gaussian kernel of 12-mm full-width at half-maximum.

### Statistical analysis

For baseline comparisons, basic characteristics and physical performance tests including knee-extension strength, OLS, and WS were compared between fallers and non-fallers using *t*-tests. Chi-square tests for differences in proportions were used to compare differences in sex and history of falling in the past year at baseline between the faller and non-faller groups. To describe variations in different physical performance factors related to falls, multivariate logistic regression analyses were performed to reveal the physical performance factors independently related to falls during the 12-month follow-up after adjusting for age, sex, body mass index (kg/m^2^), and history of falling in the past year at baseline. We calculated the odds ratios (OR) with 95% confidence intervals (CI). These statistical analyses were calculated using SPSS for Windows version 19.0 (SPSS Inc., Chicago, IL).

In the voxel-based morphometry (VBM) analysis, data preprocessing and analysis was performed with the VBM8 toolbox, which is incorporated in the SPM8 software. VBM [30] was used to examine differences in baseline gray matter volume between fallers and non-fallers. We used unpaired t-tests in SPM8 to identify the locations of smaller gray matter volume in fallers compared to non-fallers during the 12-month follow-up period using MRI data at baseline. Age and sex were included as covariates. The statistical threshold selected for these analyses was *P* < .001 (uncorrected), with an extent threshold of 100 voxels.

## Results

The characteristics and physical performance tests at baseline are presented in Table 1. Over the 12-month follow-up period, 11 of the 42 participants (26.2%) experienced at least one fall. Fallers exhibited poorer one-legged standing time (*p* < .01) and slower walking speed (*p* < .01) compared with non-fallers. In addition, the faller group had a significantly higher rate of fall history at baseline compared with the non-faller group (*p* < .01). In the multivariate logistic regression, OLS time (sec) (OR [95% CI]: 0.89 [0.81, 0.98], *p* = .02) was associated with a significantly lower rate of falls during the 12-month follow-up after adjusting for age, sex, body mass index, and history of falling in the past year at baseline. There was no statistical evidence of associations between falls and knee-extension strength (Nm) (1.02 [0.96, 1.08], *p* = .59) and walking speed (m/min) (0.91 [0.81, 1.03], *p* = .13) (Table 2).

**Table 1:** Comparison of characteristics and physical performance tests between non-fallers and fallers at baseline

	**Total (n = 42)**	**Non-fallers (n = 31)**	**Fallers (n = 11)**	**P-value**
Age, years	75.6 ± 6.3	75.2 ± 6.5	76.8 ± 5.9	0.462
Female, n (%)	18 (42.9)	12 (38.7)	6 (54.4)	0.362
History of falling in the past year, n (%)	13 (31.0)	6 (19.4)	7 (63.6)	0.006
Knee-extension strength, Nm	60.5 ± 26.8	63.4 ± 23.3	52.3 ± 34.7	0.242
One-legged standing time, sec	32.3 ± 24.2	38.9 ± 22.3	13.8 ± 19.7	0.002
Walking speed, m/m	66.7 ± 12.6	70.0 ± 11.8	57.5 ± 10.4	0.004
Mini-mental state examination, score	26.3 ± 2.7	26.6 ± 2.0	25.5 ± 3.9	0.112

**Table 2:** Multivariate logistic regression summary for physical performance on falls (n = 42)

**Variables**	**Odds ratio**	**95% confidence intervals**	***p *Value**
Knee-extension strength, Nm	1.017	0.957-1.080	0.588
One-legged standing time, sec	0.891	0.809-0.981	0.019
Walking speed, m/m	0.911	0.806-1.029	0.133

Notes: Age, sex, body mass index (kg/m^2^) and history of falling in the past year at baseline were included as covariates.

**Figure 1:**
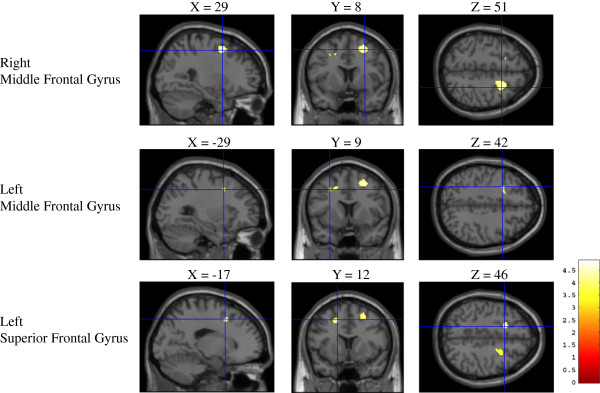
**Gray matter density in fallers versus non-fallers.** Regions of gray matter reduction in fallers compared to non-fallers (p < 0.001, uncorrected). Fallers exhibited a greater reduction of gray matter loss in the bilateral middle frontal gyrus and superior frontal gyrus.

The gray matter density profiles used for examining differences between fallers and non-fallers at baseline are shown in Figure 1. VBM analysis revealed that fallers exhibited lower gray matter density compared with non-fallers in the bilateral middle frontal gyrus and superior frontal gyrus (Table 3). These regions correspond to the premotor cortex and supplementary motor area.

**Table 3:** VBM results including age and sex as covariates

					**MNI coordinates**		
**Location**	**Cluster size (K)**	**Peak T**	**Z score**	**P (uncorrected)**	**X**	**Y**	**Z**
Right middle frontal gyrus	594	4.87	4.27	< 0.001	29	8	51
Left middle frontal gyrus	165	4.35	3.90	< 0.001	−29	9	42
Left superior frontal gyrus		4.78	4.20	< 0.001	−17	12	46

Note: *VBM* voxel-based morphometry.

## Discussion

The present study examined whether baseline physical performance and gray matter volume are related to falls during a 12-month follow-up period in community-dwelling older adults with MCI. Our results indicated that older adults with MCI exhibiting poor balance had a greater risk of falls during the 12-month follow-up period, while adjusting for age, sex, body mass index, and history of falling at baseline. In addition, baseline lower gray matter volume in the middle frontal gyrus and superior frontal gyrus was associated with the occurrence of subsequent falls. To our knowledge, this is the first study to examine the association between lower gray matter density and risk of falls in older adults with MCI.

Problems with gait and balance have been reported to have the strongest association with falling [2,31]. Slower walking speed has been found to be an independent predictor of falling [32,33]. Poor balance represented by increased postural sway and gait asymmetry has been reported to approximately triple the risk of falling [2]. A previous systematic review and meta-analysis provided a summary estimate for falls due to balance impairment at a relative risk of 1.42 [34]. Therefore, an assessment of balance and gait for older adults, particularly those without a history of falling, has been recommended [35]. Moreover, cognitive impairment has been associated with the risk of falls as well as deficits of physical function [2]. A recent systematic review and meta-analysis confirmed that cognitive deficits detected in clinical assessment are associated with an increased fall risk in community and institution-dwelling older adults [36]. A number of studies have examined the risk of falls in older adults with dementia [37]. However, little research has focused on individuals with MCI. MCI is increasingly recognized as a substantial clinical problem in older populations [38], so it is important to determine risk factors for falling among older individuals with MCI, and to develop effective fall-prevention strategies. A previous study showed that older women with MCI demonstrated a greater number of risk factors for falling compared with older women without MCI [14]. The results of the present study indicate that poor balance assessed by one-legged standing time predicts falls in people with MCI prospectively over 12 months. Although fallers exhibited slower walking speed compared with non-fallers, walking speed was not associated with the occurrence of subsequent falls after adjusting for age, sex, body mass index, and history of falling at baseline. There was no difference in the extension strength between fallers and non-fallers. The results of this study indicate that poor balance is the important factor related to an increased risk of falling among people with MCI. Muscle weakness and problems with mobility had been considered to be the important contributors to the risk of falling in older people [5], and there are presumably some relationships. In study cohorts including older people with MCI and similar lower muscle strength, like the present study, poor balance may have a greater impact on increased risk of falling than walking performance. Certainly, poor balance could be one of the predictors of walking decline among older people [39]. Balance ability may be an important dimension of physical functioning to predict the occurrence of subsequent falls among older people with MCI, as well as those with intact cognition. The present study has advantages including the examination of occurrence of subsequent falls during a 12-month follow-up period and neuroimaging assessments in older adults with MCI. However, our sample was not large, and selection bias may affect the results of the relationships between physical performance and occurrence of subsequent falls. Therefore, future studies with larger numbers of MCI subjects and a longitudinal design are needed to add evidence to the present results.

Unlike previous investigations, the current study included MRI scanning and a follow-up assessment of falls in community-dwelling older adults with MCI. The results provide the first evidence that lower gray matter volume in the middle and superior frontal gyrus is related to the occurrence of subsequent falls among older adults with MCI. Age-related changes in the brain may contribute to the subtle onset of motor disturbances in older people. Previous brain-imaging studies of older adults have reported that age-related changes in the brain, such as lower global brain volume, WMH, and microbleeds, are associated with clinical measures of poor balance and slow gait [40-43]. The association between MRI-detected lower brain volume and falls in older adults with MCI has not been examined longitudinally. In the present study, fallers exhibited decreased gray matter density compared with non-fallers in the bilateral middle frontal gyrus and superior frontal gyrus corresponding to premotor cortex and supplementary motor area. These particular regions are likely to play an important role in predicting fall-risk because the middle frontal gyrus is involved in controlling behavior with spatial and sensory guidance.

Growing evidence suggests that brain function is associated with physical function, as confirmed by neuroimaging techniques. Structural changes of the brain in older people are reported to be related to physical performance, such as gait dysfunction [44,45], postural instability [24], and lack of cardiorespiratory fitness [46]. Activation in the frontal cortex, including the premotor cortex and the supplementary motor areas, have been reported to increase during human gait by studies using near-infrared spectroscopic imaging [47-50]. Previous studies have reported that lower brain volume in the prefrontal areas is associated with slower gait in high-functioning or cognitively normal older adults [23,40,51]. Other neuroimaging studies have indicated that gait requires complex visuo-sensorimotor coordination, and is associated with activation of the medial frontoparietal region, e.g. the primary sensory and motor areas, supplementary motor area, lateral premotor cortex, cingulate cortex, superior parietal lobule, precuneus, and the infratentorial region including the dorsal region [52-54]. The middle frontal gyrus is involved in motor output and the direct control of behavior, as well as planning, spatial guidance, and sensory guidance of movement [55]. Lower gray matter volume in the premotor cortex and supplementary motor area may be risk factors for falls in older adults. Falls often occur when older individuals attempt to avoid an obstacle in their path, requiring the control of behavior and the planning of movement under sensory guidance. The premotor cortex and supplementary motor area may play an important role in preventing falls when spatial and sensory guidance are required for movement.

Several limitations of the current study should be noted. First, fall experience during the 12-month follow-up period were confirmed with two face-to-face interviews at 6-months and 12-months after baseline, while previous studies have reported that monthly fall diaries and follow-up telephone calls provide more accurate measures of fall frequency [56,57]. Second, participants who had at least one fall during the 12-month follow-up period were categorized as fallers in this study. A previous study reported that single fallers are more similar to nonfallers than to recurrent fallers on a range of medical, physical, and psychological risk factors [58]. Other studies defined fallers as people who had at least one injurious or two non-injurious falls [17,59]. In addition, our MRI scans were performed using a 1.5-T system with relatively low resolution. We performed the VBM analysis to identify the locations of group differences in gray matter volume. Therefore, we consider that our results cannot provide evidence for whether the effects of physical performance are independent of the gray matter volume or whether the latter confounds the association between the former and the fall risk. Although it is unclear whether lower gray matter volume is related to poor balance in older adults with MCI, the current study revealed that poor balance and lower gray matter volume in the middle frontal gyrus and superior frontal gyrus were associated with falls. To clarify these points, we consider that future studies including larger numbers of subjects and countable data for structural changes in the brain (e.g., described volumes in cubic millimeters) are needed.

## Conclusions

The current findings indicate that poor balance predicts falls over a 12-month period, and that lower gray matter volume in the middle frontal gyrus and superior frontal gyrus was associated with falls in older adults with MCI. Maintaining physical function, especially balance, and brain structural changes through many sorts of prevention strategies in the early stage of cognitive decline may contribute to decreasing the risk of falls in older adults with MCI.

## Competing interests

The authors declare that they have no competing interests.

## Authors’ contributions

HM has made substantial contributions to conception and design, subject recruitment, analysis and interpretation of data, and writing the manuscript. HS has made substantial contributions to conception and design, subject recruitment, interpretation of data, and writing the manuscript. TD has made substantial contributions to subject recruitment, acquisition of data, interpretation of data, and manuscript preparation. HP has made substantial contributions to conception and design, interpretation of data, and writing the manuscript. DY contributed subject recruitment and manuscript preparation. KU and KT contributed subject recruitment and acquisition of data. TLA has been involved in drafting the manuscript or revising it critically for important intellectual content. TS has made substantial contributions to conception and design and writing the manuscript. All authors read and approved the final manuscript.

## Pre-publication history

The pre-publication history for this paper can be accessed here:

http://www.biomedcentral.com/1471-2377/13/102/prepub
